# ProPhylo: partial phylogenetic profiling to guide protein family construction and assignment of biological process

**DOI:** 10.1186/1471-2105-12-434

**Published:** 2011-11-09

**Authors:** Malay K Basu, Jeremy D Selengut, Daniel H Haft

**Affiliations:** 1 J. Craig Venter Institute, Rockville, MD 20850, USA

## Abstract

**Background:**

Phylogenetic profiling is a technique of scoring co-occurrence between a protein family and some other trait, usually another protein family, across a set of taxonomic groups. In spite of several refinements in recent years, the technique still invites significant improvement. To be its most effective, a phylogenetic profiling algorithm must be able to examine co-occurrences among protein families whose boundaries are uncertain within large homologous protein superfamilies.

**Results:**

Partial Phylogenetic Profiling (PPP) is an iterative algorithm that scores a given taxonomic profile against the taxonomic distribution of families for all proteins in a genome. The method works through optimizing the boundary of each protein family, rather than by relying on prebuilt protein families or fixed sequence similarity thresholds. Double Partial Phylogenetic Profiling (DPPP) is a related procedure that begins with a single sequence and searches for optimal granularities for its surrounding protein family in order to generate the best query profiles for PPP. We present ProPhylo, a high-performance software package for phylogenetic profiling studies through creating individually optimized protein family boundaries. ProPhylo provides precomputed databases for immediate use and tools for manipulating the taxonomic profiles used as queries.

**Conclusion:**

ProPhylo results show universal markers of methanogenesis, a new DNA phosphorothioation-dependent restriction enzyme, and efficacy in guiding protein family construction. The software and the associated databases are freely available under the open source Perl Artistic License from ftp://ftp.jcvi.org/pub/data/ppp/.

## Background

Phylogenetic profiling is an established and widely known technique for inferring biological roles for unknown proteins [1]. The technique capitalizes on the propensity of proteins that work together in the same cellular system to have traveled together in evolutionary processes of speciation, gene loss, and lateral transfer. Such systems may consist of biochemical pathways, multi-subunit protein complexes, protein-modifying enzymes with their targets, *etc*. For a given protein, the evolutionary history of its family is reflected in its present day taxonomic distribution [2]. Therefore, examining protein family co-occurrence across large numbers of genomes may reveal evidence linking one protein to others that cooperate in the same system.

A phylogenetic profile in its simplest form is a series of binary characters [3], 1's or 0's that show the presence or absence of a given protein family or of some other marker across a defined set of taxonomic groups [4-6]. This profile is then scored against profiles generated from other protein families in order to find statistical evidence that the families they represent cooperate in some way. Since its inception, the technique has undergone extensive refinements [7]. Some of these improvements are: a) use of information such as logic relationships amongst proteins [8]; b) use of domains [9], single amino acid [10], or even peptides [11] instead of proteins for comparison of profiles; c) refinements in selection of reference organisms [12]; d) incorporation of evolutionary relationships of the species into the comparison of profiles [13,14]; and e) use of information such as copy numbers of domains within genomes [7]. In spite of these refinements, the technique still depends heavily on some method for approximating the boundaries of a protein family such that its members are functionally equivalent, which is a notably difficult problem to solve.

Nonetheless, phylogenetic profiling can be very successful in identifying cohorts of protein families likely to cooperate in the same cellular processes, and for making strong predictions of biological roles or molecular functions on unknown proteins when combined with other types of evidence. The technique has been used for various genetic and phenotypic traits that can be coded as binary characters [1]. Some examples are: prediction of subcellular localization of proteins [15,16]; identification of virulence associated genes in human pathogens [17]; prediction of protein domains and their interactions [9,18,19]; prediction of protein-sorting enzymes from the taxonomic ranges of their target sequences [20], prediction of restriction sites [21], etc. A few online databases now provide search and display capabilities using co-occurrence data computed through phylogenetic profiling, although the method for scoring co-occurrence is not user-adjustable. Examples include MicrobesOnline [22], STRING [23], etc. [reviewed in [24,1,25]].

The success of phylogenetic profiling is affected by considerations in four components of the method, presented here in the order in which they are applied. The first is the selection of target species. Although the effect of species selection in the success of phylogenetic profile methods is largely unexplored, it is now generally believed that the prediction accuracy increases with increasing numbers of species [12,26] while limiting taxonomic redundancy [27]. Second is the ability to find the appropriate set of homologs to a particular protein sequence in a set of genomes. This step typically relies either on prebuilt protein families, or on some methods for using sequence similarity results (e.g., BLAST) to identify members of a protein family. Third is the method of representing the phylogenetic distribution of a protein family as a profile. Although a binary vector is typical, a profile sometimes is represented by real or transformed values of raw BLAST scores [28-30,2]. Fourth, and finally, is the identification of a suitable method for scoring the match between profiles. Such methods include simple distance measures such as Hamming distances [4], mutual information content [31,32], Jaccard coefficients [33,34]; statistical measures such as Pearson's correlation coefficient [34], Fisher's exact test [14], rank correlation [2], etc. There are also methods that go beyond naïve measures and take into consideration evolution of protein families, such as tree-kernel based method [35], parsimony-based methods [36], and maximum likelihood methods [14,36,37]. Such methods, instead of considering only the distribution of protein homologs amongst the extant species, take into account the ancestors of such species and how gene gain and loss events have affected the current distribution of a protein family.

It appears that the most leverage for improvement of the method is found in the second of these: phylogenetic profiling works best when the working definition for each protein family comes as close as possible to the full set of homologs among which some specific function is conserved. Typically, however, molecular function varies within protein superfamilies, and a particular function is shared only within a narrow subset. Automated protein clustering methods or fixed sequence similarity cutoffs used for sequence homology searches cannot reliably achieve granularities that represent protein family sizes optimally. This shortfall can limit the effectiveness of available profiling methods, especially when they need to evaluate subgroups found within larger superfamilies.

We describe here ProPhylo, a distributable software framework for performing phylogenetic profiling studies. It includes the first distributable tool for performing Partial Phylogenetic Profiling (PPP), an algorithm that overcomes such limitations by optimizing protein family size simultaneously with scoring co-occurrence. Since first introducing the principle [20], we have been using PPP extensively to find key protein components of novel biological systems, and to guide construction of their corresponding protein family definitions [38-41]. We introduce as well a variation on PPP called Double Partial Phylogenetic Profiling (DPPP). DPPP starts with a single query sequence, and then optimizes protein family sizes around both the query protein and the collection of target proteins. ProPhylo provides, in addition, utilities for constructing and manipulating taxonomic profiles and for using pre-computed BLAST results to perform PPP and DPPP.

ProPhylo is a framework written in Perl and designed to perform high-performance phylogenetic profile searches using arbitrary profiles on a desktop computer. A version is also available to run on computer clusters (available on request). The software comes with a database of pre-built BLAST searches of ~1500 selected prokaryotic reference genomes for immediate phylogenetic profile search. Users additionally have the option to create their own databases. The software comes with tools for the creation and manipulation of phylogenetic profiles. At present, the software is configured to run profile searches using our own PPP algorithm, but it is designed in such a way that any scoring function can be used as a plug-in. We have plans to include several other scoring functions with future versions of the software. The software and the associated databases are freely available under the open source Perl Artistic License from ftp://ftp.jcvi.org/pub/data/ppp/.

## Implementation

### Databases

For a phylogenetic profile search, ProPhylo requires two databases: the cross-genome all vs. all BLAST search results (or a slice of this data set for at least one target genome), and the NCBI taxonomy database. The pre-formatted, ready-to-use databases can be downloaded from the ProPhylo distribution site, ftp://ftp.jcvi.org/pub/data/ppp/. The current version provides all vs. all BLAST result data for ~1500 complete prokaryotic genomes. These genomes were manually selected to remove excessive strain-level redundancy, while keeping the maximum possible taxonomic breadth. The peptide files for these genomes were downloaded from NCBI and an all *vs*. all search using NCBI BLAST was performed. Results were processed using a script supplied with ProPhylo to produce files suitable for reading by ProPhylo. These parsed BLAST result files are stored separately for each genome. BLAST results for a user-selected target genome, therefore, can be downloaded separately from other genomes, keeping the download size small.

ProPhylo depends on the NCBI taxonomy tree for extraction of phylogenetic information. Thus, a local installation of the NCBI taxonomy tree is essential to run the software. A pre-formatted SQLite (http://www.sqlite.org/) database of the NCBI taxonomic tree can be downloaded from the ProPhylo software distribution site. Alternatively, scripts supplied with the package can automatically download the NCBI taxonomic tree from the NCBI website and create the necessary SQLite database on the user's desktop machine.

As an alternative to using the supplied databases, users can create custom databases. An extensive collection of scripts are supplied with software package, including scripts for performing BLAST searches and parsing BLAST result files, to create the databases suitable for use with ProPhylo.

### Profile creation

ProPhylo at present uses profiles in binary form, as a tab-delimited text file containing two columns: a taxonomic ID as defined by NCBI taxonomy database followed by a 1 and 0 to indicate the presence or absence of a trait in the corresponding genome. A user can create such a profile file from several sources. Scripts supplied with the package can create a query profile from BLAST, from HMM search results, or even from a collection of sequence identifiers. A user also has the option of supplying a manually created or modified profile file; taxa may be toggled between 0 and 1, or may be removed entirely from influence on PPP results by omission from the query profile.

### Creation of profiles used in this study

TIGRFAMs [42] model TIGR03185 describes the DptD (previously DndD) protein that is one of the four trustworthy markers for the presence of the DNA phosphorothioation (DPT) system [43]. Examination of the 80 proteins (or protein fragments) that score > = 200 to this model using HMMER3 [44] identifies 70 different sporadically distributed genomes from the 1466 genomes searched (4.8%) and was used to create the DPT profile. The methanogenesis profile was created manually to agree with the scientific literature.

### Utility scripts

For effective use of the phylogenetic profile methods, providing only the method to compare profiles is not enough. The software package comes with a set of programs (Additional file 1 Table S1) to effectively manage and manipulate profiles. The programs allow the user to perform the following operations on profiles:

1. The user can use scripts to manipulate profiles and perform several operations on profile files, such as Boolean and set operations on pairs of profiles to create new profiles. The operations make it possible to define profiles for working with orphan markers, or with recurring pathway holes.

2. The user can create a profile directly from the NCBI taxonomic database. These profiles can be used to query for proteins most universally conserved within a lineage. They can also be used, through Boolean operations, to perform taxonomic restrictions on other profiles. For example, using a profile based on a marker for pathway X, the following question is easy to ask: what proteins occur only in the Proteobacteria, and only when pathway X is present?

3. The user can perform masking operations that remove genomes whose assignments of the query trait are uncertain or irrelevant and a possible source of noise. This masking makes it possible to contrast species with all three markers A, B, and C for some system against species with none of the three, while ignoring species whose incomplete systems reconstructions may represent decaying systems, faulty classifiers, pathway variants, or other confounding situations.

### Profile search

At present, the ProPhylo package only supports comparison using the Partial Phylogenetic Profile (PPP) algorithm [20]. The algorithm is called "partial" because it does not score two full profiles (each equal in size to the number of genomes in the all-vs-all data set) against each other. Instead, at each step in an optimization procedure it examines the partial profile that consists only of genomes encountered so far while descending a list of best BLAST hits. It assigns all of these genomes the value 1, and scores each such partial profile for consistent agreement with the query profile, which contains both matching (1) and mismatching (0) values. In order to compare different proteins in a genome by their relative ability to match the query profile, each protein is scored based on the depth in its list of best BLAST hits where its match to the query profile shows the greatest statistical significance.

### Algorithms

A profile *P *= (*P_1_*,....*P_n_*) ∈ {0,1}^*n *^is defined over a collection of taxonomic identifiers *T *= {*t_1_*,...,*t_n_*}, with negative examples being *T*_0 _= {*t_i_:P_i _= *0} and positive examples being *T*_1 _= {*t_i_:P_i _= *1}. We iterate through each gene in a genome. For each gene (*G*) we get its BLAST result (*R*), which is a collection of tuples (*g_j_*,*s_j_*) for *j *= 1,...,*m*, where *g_j _*is a GenBank identifier (GI) and *s_j _*is a measure of significance, where only the most significant match from each taxon is considered. For each such *R*, we iterate through the GIs sorted in decreasing order of significance. For each *g_j _*we get its taxonomic ID, *u_j_*∈*T *While iterating over *j *= 1,...,*m *the algorithm calculates,

$$p_{j}=\overset{j}{\underset{k=h_{j}}{\sum }}\left(\begin{array}{c} j\\ k \end{array}\right)p^{k}{\left(1-p\right)}^{k},$$

where, *h^j ^*= |{*u*_1_,...,*u_j_*}∩*T*_1_|, i.e., the number of profile's positive genome seen up to index *j*. The value *p_j _*is, therefore, simply the probability, according to the binomial distribution, of finding at least *h_j _*hits (taxonomic identifiers whose value is 1 in the query profile) after *j *distinct taxa have been encountered in the BLAST hits list. The parameter *p *is the prior probability either provided by the user or calculated as the relative frequency of positive taxa in the input profile: *p *= |*T*_1_|/(|*T*_0_|+|*T*_1_|)

The score of a particular gene *G *is then reported as p* = min*p_j_*, that is, the most significant probability computed for any depth in the BLAST hists list. For a genome, the results are presented as a sorted order of scores for each gene along with the values of p*, and its corresponding *j *and *h_j_*.

A variation of the algorithm is double partial phylogenetic profiling (DPPP). This allows iterative searching starting with only a single given sequence. The algorithm iteratively goes down the BLAST hit list of a given protein, creating a new version of the taxonomic profile each time it finds a new taxon. These sets of query profiles are then searched using the PPP algorithm described above. The significant hits for each search are then collected and a sorted list of hits is generated as output. DPPP automates the generation of the optimized PPP data sets needed to guide systems biology discovery and protein family construction. However, presently we do not provide any post processing tools to automate building protein families according to DPPP results.

To accelerate the speed of the search we developed several heuristics, such as pre-calculation of the maximum binominal probability given a particular profile. A read-ahead mechanism determines whether it is possible to improve on the existing best score, given the remainder of the hit list, and stops the search if there is no chance of such improvement.

The sensitivity of the algorithm can also be modulated at the user's discretion. One such mechanism is collapsing terminal branches of the taxonomic tree. The software fully understands the NCBI taxonomic tree; a search can be performed using higher taxonomic levels such as genus or family rather than the strain-level identifier from NCBI taxonomy tree. The algorithm will walk up the taxonomic tree to the level specified by the user and count that node only once for scoring. Another user option is to override the default calculation of *p*; increasing this value increases the penalty for mismatches to the query profile and thus helps to find only-if relationships even for proteins that are relatively rare.

### Software Details

ProPhylo is written in the Perl computer language and, therefore, theoretically can run on any platform where Perl is available, however at present we support only MacOSX and Linux officially. The software runs searches in parallel taking full advantage of multi-core processors frequently available in modern desktops. The software also comes in a flavor that runs on compute clusters (available on request).

In addition to a collection of scripts for various tasks related to phylogenetic profile comparison, ProPhylo contains an object-oriented Perl library. Components of the search mechanism, such as BLAST results, phylogenetic profiles, algorithms for comparison of profiles, are represented as software classes and thus are extensible and readily modifiable. One advantage of such a design is apparent in the case of the implementation of the algorithm modules, with each algorithm being a plug-in. Any new algorithm, currently not supported by ProPhylo, can be easily supplied by inheriting from an interface and providing the concrete implementation of a few virtual methods.

The user can download the software and the required databases from the distribution site. Detailed documentation is provided with the package for correct installation of the package and the database. The user also has the option to either create the databases on local machine or to use the pre-made databases available on the FTP site.

## Results

### PPP method overview

**Figure 1:**
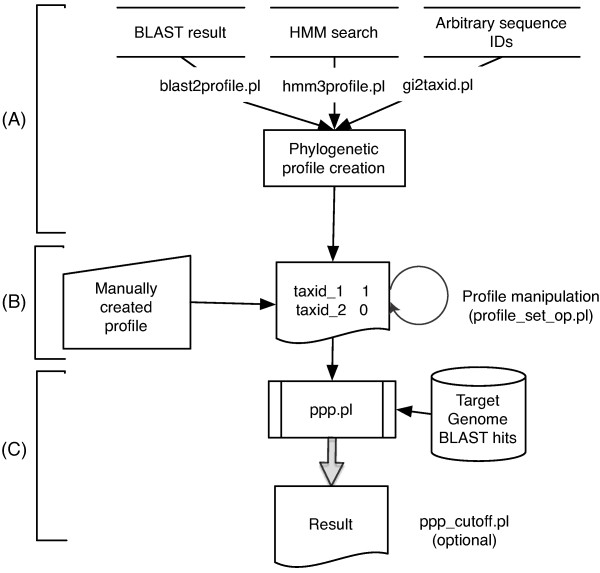
**Flowchart for profile search using ProPhylo with the Partial Phylogenetic Profile algorithm (PPP)**. For each step, the relevant software name is indicated. (A) Creation of a profile from various search methods or directly using a set of GenBank GIs. (B) The created profile is a tab delimited text file containing a set of taxonomic IDs from NCBI taxonomic database and 1's and 0's for the presence and absence of the query protein family. (C) The main script ppp.pl searches a given genome using the query profile and generates results as a ranked list of candidate functionally linked proteins.

The workflow of phylogenetic profile searching using ProPhylo with the PPP algorithm is shown in Figure 1. A profile search begins with BLAST or HMM results, or even from a collection of GIs (Figure 1A). A script then determines the taxonomic distribution of the GIs in the hits list file and creates a query profile (Figure 1B). A user can also provide a profile directly for the search. The user may also optionally provide a value to override the calculated binomial probability factor, *p *(see detailed discussion below), and/or specify a taxonomic level at which to filter the profile. Then, given a target genome to search and the query profile, the software iterates through every protein in the target genome, finding that protein's optimal correlation to the query profile. It orders its results by the statistical significance of each individually optimized score, and reports the numbers of matches and mismatches to the profile for each protein where its optimum was found (Figure 1C).

### Finding molecular markers of methanogenesis - a large-scale test

An example that illustrates the use and utility of PPP is the identification of universal molecular markers of methanogenesis. Methanogenesis requires a number of unusual cofactors that are rare or unknown outside the methanogens. All methanogens belong to the archaea, but they are quite divergent. Furthermore, additional non-methanogenic lineages such as *Archaeoglobus *and *Ferroglobus*, rather than methanogens exclusively, descend from the presumed last common ancestor to all methanogens. Finally, methanogens are metabolically varied. Some are able to convert only methanol, and not carbon dioxide, into methane, and the group includes both thermophiles and mesophiles.

In the TIGRFAMs collection of protein family definitions [42], we have been able to construct thirty-three manually reviewed HMMs serving to identify protein families whose membership is universal in, yet limited to the archaeal methanogens among the current available set of prokaryotic reference genomes. This is a large set of proteins to associate with a single biological process; it serves as a positive control set while demonstrating how widely sequence similarity cutoffs must be varied to choose protein family boundaries correctly. The method was performed on a test genome, that of *Methanohalophilus mahii *DSM 5219, using a literature-based methanogenesis query profile in which 28/1466 genomes were marked 1 (methanogen) rather than 0 (non-methanogen).

**Figure 2:**
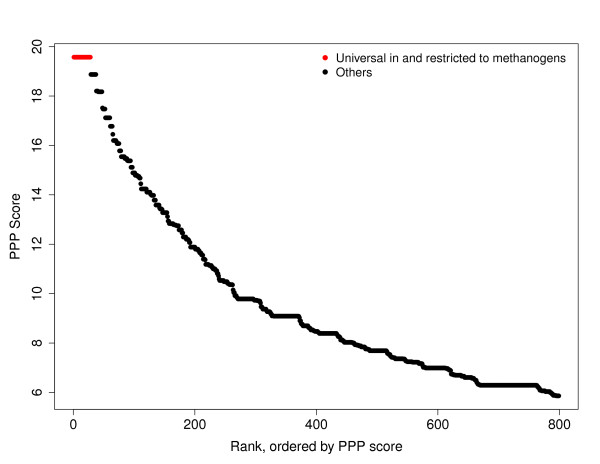
**Distribution of the scores of PPP**. The query profile contains all methanogens marked as 1, and the target genome is *Methanohalophilus mahii *DSM 5219. The binomial distribution probability parameter is raised to 0.2, which helps proteins absolutely restricted to the methanogens, although not universal among them, to get a better relative rank. The plot shows PPP score on the Y-axis, and rank, sorted by PPP score, on the X-axis. The top-scoring 28, with perfect agreement to the query profile, are colored red.

In the PPP results, the top-scoring twenty-nine proteins (Table 1 Figure 2) were those found to occur universally in and yet restricted to the methanogens: the first 28 species encountered in the BLAST lists were the 28 methanogens in the profile. This allows an appropriate cutoff score for BLAST results to be selected to define the families. The table shows in the second column how high (permissive) the E-value had to become to bring in the last methanogen genome, and in the third column the E-value of the first hit outside the set of archaeal methanogens. The list includes proteins from seven consecutive genes for subunits of tetrahydromethanopterin S-methyltransferase, and eight consecutive genes from the methyl-coenzyme M reductase region. The list contains another long gene cluster, encoding six different uncharacterized proteins (which had already been recognized as methanogenesis markers and captured as TIGRFAMs models, see below) and the methyl coenzyme M reductase system component A2. Conspicuously absent from the list are proteins of essential processes such as protein translation, whose presumed high barriers to gene loss and lateral transfer make them popular choices for computing species phylogenies. The ~200 sequences ranked highest by PPP score (Figure 2) is highly enriched in proteins whose functions are linked to methanogenesis, although not necessarily exclusively or universally. No ribosomal proteins, tRNA ligases, translation factors, cell division proteins, etc., occur until the 84^th ^best hit by PPP to the methanogenesis profile.

**Table 1:** Proteins identified in *Methanohalophilus mahii *DSM 5219 as PPP's top hits with perfect agreement (28 of 28 genomes) to the methanogenesis phylogenetic profile, showing BLAST E-values flanking the boundaries selected by PPP.

GI number	Last True	First False	Protein Functional Assignment
294495086	1e-24	4e-23	paralog of MtrA
294495087	5e-52	2e-46	paralog of MtrH
294495289	5e-170	2.5	methyl-coenzyme M reductase, alpha subunit
294495290	8e-68	0.11	methyl-coenzyme M reductase, gamma subunit
294495291	5e-35	(none)	methyl-coenzyme M reductase operon protein C
294495292	7e-17	0.63	methyl-coenzyme M reductase operon protein D
294495293	2e-130	2.4	methyl-coenzyme M reductase, beta subunit
294495294	5e-94	3e-07	methanogenesis marker 10 radical SAM protein
294495889	0.38	0.64	protein of unknown function DUF2098
294495924	7e-131	6e-130	formylmethanofuran dehydrogenase, subunit A
294495926	6e-08	1e-07	formylmethanofuran dehydrogenase, subunit D
294496062	2e-73	4e-09	methanogenesis marker 13 metalloprotein
294496216	9e-68	3e-55	methanogenesis marker 2 protein
294496423	5e-48	(none)	tetrahydromethanopterin S-methyltransferase, MtrE
294496424	3e-25	(none)	tetrahydromethanopterin S-methyltransferase, MtrD
294496425	1e-18	0.78	tetrahydromethanopterin S-methyltransferase, MtrC
294496426	0.003	0.48	tetrahydromethanopterin S-methyltransferase, MtrB
294496427	2e-37	8e-19	tetrahydromethanopterin S-methyltransferase, MtrA
294496429	4e-04	0.40	tetrahydromethanopterin S-methyltransferase, MtrG
294496430	8e-76	2e-48	tetrahydromethanopterin S-methyltransferase, MtrH
294496497	9e-52	0.45	methanogenesis marker 7 protein
294496498	0.011	0.36	methanogenesis marker 17 protein
294496499	1e-115	9e-31	methanogenesis marker 15 protein
294496500	3e-34	1.5	methanogenesis marker 5 protein
294496501	2e-14	3.0	methanogenesis marker 6 protein
294496502	2e-45	1.8	methanogenesis marker 3 protein
294496503	1e-144	4e-39	methyl coenzyme M reductase system, AtwA protein
294496608	2e-43	9e-31	paralog of AtwA
294496619	2e-72	0.031	methanogenesis marker 14 protein

In Table 1 two of the proteins found are subunits A and D of formylmethanofuran dehydrogenase (FMD), part of a C1 transfer pathway required by methanogens but not absolutely restricted to them. These two are, from one perspective, false-positives, in the sense that a complete family of functionally equivalent FMD proteins would include members from more species than just the methanogens. On the other hand, within the methanogens these proteins do act in methanogenesis, and PPP is accurately identifying that branch of the family as a universal marker of methanogenesis.

PPP performs an excellent enrichment for proteins directly involved in methanogenesis in the set of proteins earning top score. Thirty-four of the top forty genes returned by PPP are members of previously identified methanogenesis marker families built in TIGRFAMs and identified within Genome Properties [42]. These are mixed with six genes whose BLAST lists generate patterns deviating from the methanogenesis profile by the absence of only one genome or the presence of one extra genome. Five representatives of the TIGRFAMs/Genome Properties marker set have similar off-by-one patterns, one of which can be explained by a missed gene call, and another by a truncated gene that may be a sequencing or assembly error. Including the remaining three off-by-one cases, a total of eight of the marker genes have imperfect profiles. This is by no means unexpected as the use of raw BLAST lists as input is undoubtedly noisy (see below). It is clear, however, that using a query profile based on a phenotypic trait, or a molecular marker used to signify such a trait, can provide a highly significant enrichment of proteins directly involved in the process in the set of proteins with the best scores by PPP.

### From BLAST to HMM: ProPhylo to guide protein family construction

The core computational function in PPP picks a score cutoff for a sorted list of BLAST hits in order to optimize a working definition of a protein family, based on which the family's match to the query profile can assessed. BLAST, however, suffers certain limitations. First, there is a limit in sensitivity. Second, in any protein family, both heterogeneity in the rates of evolution in different lineages and regions of unusual sequence composition can interfere with the ability of the evolutionarily most closely related proteins to outscore more distantly related proteins. Limitations of BLAST explain the absence of the MtrF protein, tetrahydromethanopterin S-methyltransferase F subunit (GI:294496428) from the list of proteins in Table 1 that perfectly match the methanogenesis query profile. Two archaeal methanogens have MtrF proteins divergent enough not to show up in the BLAST hits list for GI:294496428, letting that protein rank only the 42^nd ^best hit by PPP.

HMMs based on protein multiple sequence alignments regularly outperform BLAST, both in sensitivity and in the discrimination of protein family members from non-members. An HMM (TIGRFAMs model TIGR02507) made from the alignment of just three MtrF sequences, for example, finds the complete MtrF family without a false positive. Constructing tools such as model TIGR02507 that cleanly identify molecular markers in large data sets often is a goal when phylogenetic profiling is performed. The ProPhylo software package, therefore, provides the utility **ppp_hmmer.pl **for analyzing HMMER3 search results files. It uses the compute engine of PPP, reading GI numbers ordered by HMM score rather than BLAST score. It finds and reports an optimal depth in an HMM hits list for matching a query profile, which aids in setting proper score cutoffs. This utility should be useful in workflows that begin with PPP search results and aim to develop new HMM-based protein family identification rules.

### PPP with user-controlled scoring behaviors - discovering novel DNA phosphorothioation-dependent proteins

PPP results show candidate protein families whose correlations to the query profile outscore the background from uninteresting correlations, as from similar taxonomy (all methanogens are archaeal) or from similar environmental pressures (most organisms that need to synthesize histidine also need to synthesize tryptophan). A rare protein meaningfully correlated (*e.g*. absolutely restricted) to another can outscore the background if an appropriate scoring system is used. PPP scoring depends on the binomial distribution; changing its parameter *p *will determine which scores better, 25 "YES" genomes out of 75, or 7 out of 7. A command line switch that forces use of a different probability *p *from what is calculated naively from the query profile can steer a PPP search toward better detection of certain types of relationships. Using a larger value of *p *increases the effective penalty for each mismatch, and makes it easier to detect even rare proteins that approximate an "only if" relationship to the query profile. Figure 2 shows an example of using a binomial distribution probability parameter raised from 0.02 to 0.2 to achieve a low background in PPP results based on the methanogenesis query profile. (Conversely, a smaller value of *p *lowers the penalty and steers PPP towards the identification of families absolutely including the set described by the profile, but also including genes from other genomes.)

The DNA phosphorothioation (DPT) system presents a challenge to phylogenetic profiling that can be addressed by application of this technique. The DPT system catalyzes a post-replication sulfur modification of the phosphate backbone of DNA, first identified in *Streptomyces lividans *[45]. The proteins DptF, DptG, and DptH are now known to represent the first restriction enzyme system specific for DPT modification sites [46]. There is no particular reason why all organisms with the DPT system should necessarily encode this particular restriction system, and in fact, DptFGH are only observed in a strict subset of those genomes. For example, a DPT profile based on the DptD gene (see Methods) yields a profile with 4.8% "YES" genomes (*p *= 0.048). Artificially raising *p *approximately 5-fold to 0.25 and running PPP on the genome of *Pseudoalteromonas haloplanktis *TAC125 yields the DptFGH genes as the top hits after the components of the DPT system itself. This is despite the DptFGH genes being present in roughly 1/4 of the genomes encoding the DPT system. Additional, yet-uncharacterized DPT-dependent systems should be expected having distinct subset distributions, and some of these may be relatively rare. PPP can be used with a raised value of *p *to enrich its results for protein families that are subsets of the DPT profile and likely candidates for such systems.

A PPP search of *Serratia odorifera *4Rx13, using the DPT profile with *p *set to 0.4 yields, in addition to the DPT system components and the DptFGH genes, the protein GI:270263649 despite its occurring in only seven genomes (Table 2). With the naïve value of *p *at 0.048 it is only the 34^th ^best hit. The seven homologs of this protein are invariably found adjacent to the elements of the DPT system; sometimes the DptFGH genes are present, sometimes not. It would appear that the homologs of protein GI:270263649, do represent a novel DPT satellite protein family, analogous to DptFGH. This family is now represented by the TIGRFAMs model TIGR04172. Guided by PPP similar results generated with elevated *p *values, we have constructed TIGRFAMs definitions for several additional strictly DPT-dependent families. TIGR04062 describes an uncharacterized protein (n = 25), while TIGR04095 describes a subfamily of PF04851, "type III restriction enzyme res subunit," (n = 26), apparently the second discovery of a DPT-dependent restriction enzyme. Both of these families include members located adjacent to DPT system genes.

**Table 2:** Top hits by PPP in *Serratia odorifera *4Rx13 using a query profile for DptD (TIGR03185 family) of the DNA phosphorothioation system, with a modified probability of 0.4.

**GI number**^**1**^	DPT **Genomes**^**2**^	Total **Genomes**^**3**^	**Score**^**4**^	Protein Functional Assignment
270263651	66	66	26.26	DptD (DndD) (DNA phosphorothioation)
270263652	59	59	23.48	DptC (DndC) (DNA phosphorothioation)
270263653	32	32	12.73	DptB (DndB) (DNA phosphorothioation)
270263650	19	19	7.56	DptE (DndE) (DNA phosphorothioation)
270263648	16	16	6.37	DptH (DPT-dependent restriction)
270263645	18	20	5.30	DptF (DPT-dependent restriction) (N-terminal)
270263647	15	16	4.97	DptF (DPT-dependent restriction) (C-terminal)
270263646	12	12	4.78	DptG (DPT-dependent restriction)
270263649	7	7	2.79	DGQHR domain protein

^1^NCBI GI. ^2^The number of unique taxa common to the query profile and the target BLAST hit list. ^3^The total number of unique taxa in encountered where PPP found the optimal depth. ^4^Score reported by PPP as the negative logarithm of the P-value at the optimal depth, which is lowest that can be obtained from the binomial distribution for any depth.

### Double-Partial Phylogenetic Profiling (DPPP)

**Figure 3:**
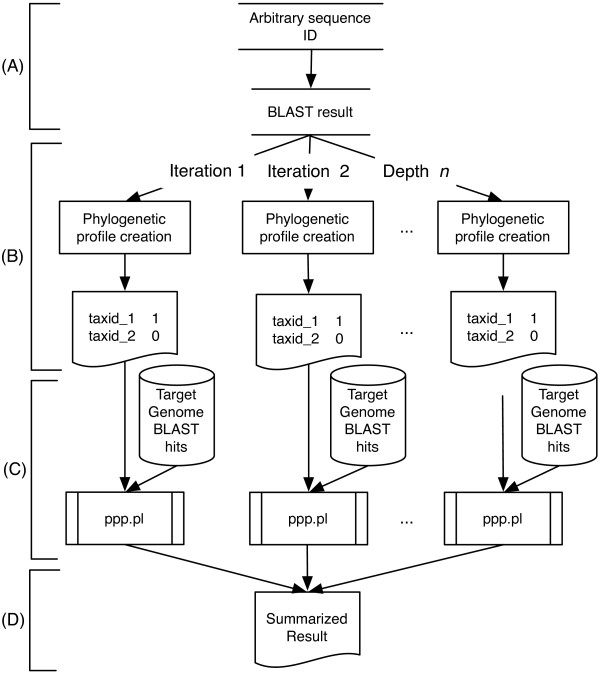
**Flowchart of profile search using ProPhylo with Double Partial Phylogenetic profiling (DPPP)**. (A) Search begins with a single query sequence with its BLAST hits. (B) The program then generates a different query profile for each depth in the BLAST hit list. (C) Each of these profiles is then searched against the target genome. (D) The top hits for each of these searches are then collected and the output is presented sorted by significance.

In the TIGRFAMs collection of protein families, models that describe equivalogs (proteins conserved in function since their last common ancestor) can vary in sizes, ranging from universal (100%) to less than 1% in coverage of prokaryotic reference genomes. When building a protein family to represent one component of a new and previously unknown complex system, the proper protein family size cannot be known in advance. DPPP addresses this limitation by taking a query protein and systematically varying the numbers of its closest matches by BLAST as the basis for constructing query profiles for use in PPP (Figure 3). If a protein participates in a multicomponent system, then for the query protein, the BLAST hits list depth that comes closest to matching the list of genomes that actually have the system should generate the best query profile. For the other proteins in the system, that query profile should provide their best PPP scores.

**Figure 4:**
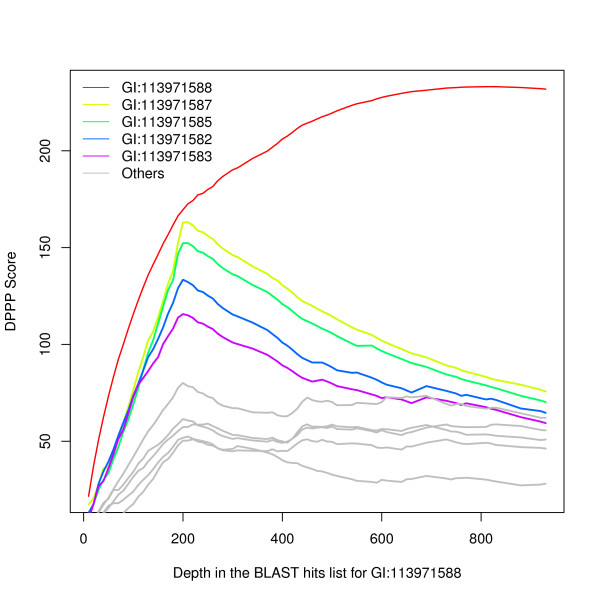
**Double partial phylogenetic profiling (DPPP) using the GTP-binding protein HydF (GI:113971588) as query**. For the query protein (red), the curve rises monotonically, because it measures the correlation of the list of species it generates to itself. Among all proteins other than the query, the peak score for any protein occurs (for HydE, GI:113971587,) where the query protein BLAST hits list depth is about 210. DPPP scores are shown for query protein depths 10 to 930, sampled every tenth hit, for the ten proteins that scored the best at the depth 210. The curves for HydE (GI:113971587, olive), HydG (GI:113971585, green), and the hydrogenase large (GI:113971582, blue) and small (GI:113971583, purple) subunits all peak at this query protein depth, which largely exhausts the list of species that carry the [FeFe] hydrogenase maturation system (see text and Table 3 for details). Proteins unrelated to [FeFe] hydrogenase maturation are shown in gray.

The [FeFe] hydrogenase H-cluster maturation GTPase HydF [47], GI:113971588 from *Shewanella sp*. MR-4, was selected as an example. DPPP searched a range of depths in the hits list to the query protein, from 10 genomes to 930 and the best score for any protein other than itself occurred when the BLAST score cutoff gave a query profile with 200 "YES" genomes of the 1466 in ProPhylo (Figure 4). Results for the top ten proteins using this depth are presented in Table 3; proteins proposed to belong to the system of [FeFe] hydrogenase maturation are in boldface. The maturase proteins HydEFG perform best followed at a somewhat lower score by the hydrogenase large and small subunits (because genomes from a few species encode [FeFe] hydrogenase genes but lack the three maturases).

**Table 3:** Top 10 hits from DPPP at in *Shewanella sp. *MR-4, with the [FeFe] hydrogenase maturation GTP-binding protein HydF (GI:113971588) as query, at a query protein BLAST hits depth of 210 (finding 200 genomes)

**GI number**^**1**^	**YES genomes**^**2**^	**Total genomes**^**3**^	**BLAST depth**^**4**^	**Score**^**5**^	**HMM-based protein annotation**^**6**^
**113971588**	**200**	**200**	**209**	**172.61**	**[FeFe] hydrogenase H-cluster maturation GTPase HydF (TIGR03918)**
**113971587**	**189**	**189**	**206**	**163.11**	**[FeFe] hydrogenase H-cluster radical SAM maturase HydE (TIGR03956)**
**113971585**	**179**	**180**	**187**	**152.29**	**[FeFe] hydrogenase H-cluster radical SAM maturase HydG (TIGR03955)**
**113971582**	**191**	**218**	**485**	**132.19**	**iron hydrogenase, large subunit (TIGR02512)**
**113971583**	**159**	**176**	**272**	**115.03**	**iron hydrogenase, small subunit (PF02256)**
113970224	194	336	613	78.43	thiazole biosynthesis protein ThiH (TIGR02351)
113971167	169	320	480	60.69	hydroxylamine reductase (TIGR01703)
113971205	181	374	603	57.37	radical SAM protein (TIGR01212)
113971473	161	327	484	52.43	peptidase, U32 family (PF01136)
113971082	132	240	843	50.34	MATE domain protein (PF01554)

Proteins related to [FeFe]-hydrogenase are marked in boldface.

^1^NCBI GI. ^2^"YES" denotes number of unique taxa common to query profile and the target BLAST hit list. ^3^The total number of unique taxa found in the target BLAST list at the level that gives the best PPP score. ^4^The depth in the target BLAST list that give the best PPP score, which may reflect multiple hits in some genomes. ^5^Score reported by PPP as the negative logarithm of P-value. ^6^TIGRFAMs and Pfam models were used because existing GenBank annotations were out of date.

**Figure 5:**
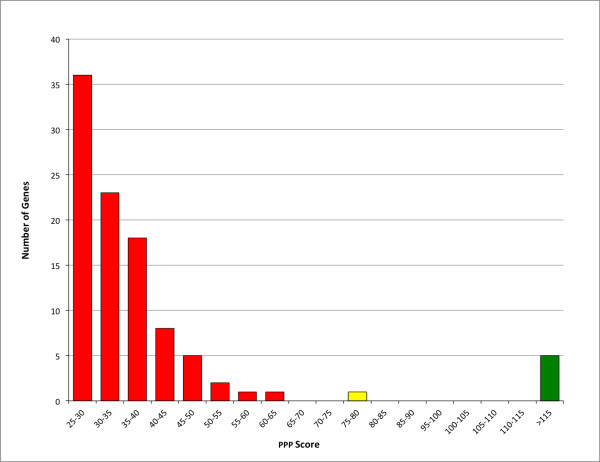
**Distribution of PPP scores resulting from a GI:113971588-based query profile at a depth of 200 distinct genomes (Shewanella sp. MR-4)**. Green: the query gene itself and the four correlated hydrogenase and hydrogenase maturation genes listed in Table 3. Yellow: the ThiH gene.

The sixth protein listed in Table 3 is ThiH, a radical SAM protein for thiamin biosynthesis. Its PPP score, 78.4, is well above background (the highest scores seen for protein families with no relation to hydrogenase maturation, Figure 5), but this is misleading. ThiH is closely related to HydG. At a depth of 613 proteins in its BLAST results, the mix of ThiF and HydG proteins represents 336 genomes, of which 194 have the GTP-binding protein HydF. This illustrates that PPP results should be read with additional caution for any protein that is homologous to a better-scoring protein from the same genome.

ProPhylo offers the user a flexible environment for performing phylogenetic profiling. It not only supports the discovery of cohorts of proteins that work in concert, but also guides initial steps in protein family construction by reporting a meaningful suggested protein family size. Its computation is based on the principle that the correct granularity for defining individual protein families may not be known in advance. In fact, we have shown here that the "right" BLAST score E-value cutoff can vary over 130 orders of magnitude (Table 1), depending greatly on protein length and the degree of conservation in the families in question. The search strategy is analogous to that of BLAST, which ranks all proteins in a target database on the basis of each protein's best possible pairwise alignment to the query. PPP scores every protein in a genome against the query profile by first finding that protein's best possible family size, and therefore best score.

Investigators using PPP may choose runtime parameters, or design query profiles, to favor particular avenues of inquiry. Query profiles can represent not only phenotypes or protein families, but various other possible starting points for research investigations: pathway holes, short motifs found on proteins in some genomes but not others, genomes with known molecular markers in unexpected contexts, etc. The flexibility in the way a query profile can be defined and how it is used, combined with the software's ability to base its searches on optimized working definitions for protein families, makes the ProPhylo package a powerful investigational tool in comparative genomics.

The examples presented here illustrate the potential of profiling analysis using PPP and DPPP and some of the intrinsic limitations of the method. Results are very much dependent on the nature of the phylogenetic profile under study. The methanogenesis profile is very similar to a "taxonomic" profile, one capturing every organism in the test set descendant from a common ancestor. Such an information-poor profile can generate an enriched list of marker genes, but inhomogeneities in similarity scoring by BLAST, and infrequent data errors, serve to blur the separation between genes whose function is directly linked to the process represented by the profile, and those matching by chance or indirect relationships. However, given sporadic, information-rich profiles that are characterized by a greater degree of gene loss and lateral gene transfer (differing widely from the taxonomic background), significant signal-to-noise can be realized. For instance, the high information content of the DNA phosphorothiolation profile enables the identification of real relationships even when the overlap between profiles is very small, by using ProPhylo's ability to modulate the expected probability factor, *p*. Even in cases such as the HydF example, where the distribution of a functionally homogeneous protein family is not posited in advance, the use of DPPP can extract both the correct family scope and the identities of process-linked partners given sufficient information in these patterns.

## Conclusion

ProPhylo is a software package for high-performance phylogenetic profile searches on desktop computers. The package implements Partial Phylogenetic Profiling (PPP) and Double Partial Phylogenetic Profiling (DPP), algorithms to facilitate protein family construction using phylogenetic profiles. The computational advances represented by the PPP and DPPP algorithms have the potential to take phylogenetic profiling beyond the limited correlation of pre-formed protein families, and to remove much of the insensitivity of previous methods due to the vagaries of protein cluster calculations. With ProPhylo, users will be able to generate and modify custom profiles, iterate and refine hypotheses, and tune parameters to perform real-time profiling research in a manner distinctly more powerful than is currently available.

## Availability and Requirements

Project name: ProPhylo; Project home page: ftp://ftp.jcvi.org/pub/data/ppp/; Operating system(s): Platform independent; Programming language: Perl; Licence: Open Source Perl Artistic License.

## Authors' contributions

MKB created the ProPhylo software and the associated databases. JS implemented prototype versions of PPP algorithms. DH performed the biological investigations. All authors made contributions in improving the algorithms, writing the manuscript and read and approved the final manuscript.

## Supplementary Material

Additional file 1 **Supplementary Table 1**. Table of the utility scripts and their uses, supplied with ProPhylo software. Click here for file
