# Supplementary material for: ProPhylo: partial phylogenetic profiling to guide protein family construction and assignment of biological process
Source: BMC Bioinformatics. 2011 Nov 9;12:434. doi: 10.1186/1471-2105-12-434 (PMC3226654; doi:10.1186/1471-2105-12-434)
Supplement: Additional file 1 — Supplementary Table 1. Table of the utility scripts and their uses, supplied with ProPhylo software. [file 1471-2105-12-434-S1.DOC]

**Supplementary Table S1:** The list of the utility scripts and their uses, supplied with ProPhylo. Only the scripts relevant for PPP and DPPP searches are listed.

| Script name | Path | Purpose |
| --- | --- | --- |
| gi_profile.pl | bin/ | For each GI number in a file, attaches the state 1 or 0 for its genome in a query profile |
| gi2taxid.pl | bin/ | Gives the taxonomic ID of a given GI. |
| hmm2profile.pl | bin/ | Creates a profile out of a HMMER2 search result. |
| hmm3profile.pl | bin/ | Creates a profile out of a HMMER3 search result. |
| ppp_cutoff.pl | bin/ | Given a raw PPP result file, marks the file for significant hits using a slope detection parameter. |
| ppp_hmmer.pl | bin/ | Scores a profile against a HMMER3 result file using PPP algorithm. |
| profile_set_op.pl | bin/ | Performs set operations on profile files. |
| profile_tax_filter.pl | bin/ | Filters a profile by given taxonomic IDs. |
| taxdist_fasta.pl | bin/ | Creates taxonomic distribution from a FASTA file. |
| cache_blast_result.pl | util/ | Creates the PPP database folder structure from a BLAST result file for local search using PPP. |
| create_description_db.pl | util/ | Creates the database for sequence descriptions from a FASTA file with descriptions in the definition line. |
| *update_taxonomy.pl | SeqToolBox/install/ | Automatically downloads taxonomy data from the NCBI website and creates a local database. |

*Part of a separate software distribution, SeqToolBox (Basu MK, unpublished).
